# Protective Effects of Safranal Against Spike Protein-Induced Mitochondrial Dysfunction and Inflammation in Peripheral and Central Immune Cells

**DOI:** 10.1016/j.cdnut.2025.107629

**Published:** 2025-12-25

**Authors:** Antonella Girgenti, Martina Letizia Contente, Miriam Buttacavoli, Laura Palumbo, Flores Naselli, Sabrina Dallavalle, Gigliola Borgonovo, Pasquale Picone, Andrea Pinto, Domenico Nuzzo

**Affiliations:** 1 Institute for Biomedical Research and Innovation, National Research Council of Italy, Palermo, Italy; 2 Department of Food Environmental and Nutritional Sciences, University of Milan, Milan, Italy; 3 Department of Biomedicine, Neuroscience and Advanced Diagnostics, University of Palermo, Palermo, Italy; 4 Department of Biological, Chemical and Pharmaceutical Sciences and Technologies, University of Palermo, Palermo, Italy

**Keywords:** SARS-CoV-2 Spike protein, neuroinflammation, mitochondria, safranal, neuroprotection

## Abstract

**Background:**

Saffron (*Crocus sativus L*.) contains bioactive molecules with antioxidant, anti-inflammatory, and neuroprotective properties. Growing evidence indicates that severe acute respiratory syndrome coronavirus 2 (SARS-CoV-2) promotes neuroinflammation and mitochondrial dysfunction contributing to neuro-coronavirus disease.

**Objectives:**

The aim of this study is to evaluate the antioxidant, anti-inflammatory, and neuroprotective effects of 3 saffron derivatives, picrocrocin, 4-hydroxysafranal, and safranal, in peripheral immune cells and microglia, and to test the hypothesis that these compounds, especially safranal, counteract Spike protein 1(S1)-induced inflammation and mitochondrial dysfunction.

**Methods:**

An immortalized murine microglial cell line (BV2) and human peripheral blood mononuclear cells (PBMCs) from healthy donors were treated with saffron derivatives at nontoxic concentrations (0.05–0.5 mM). Cytotoxicity (3-(4,5-dimethylthiazol-2-yl)-5-(3‑carboxymethoxyphenyl)-2-(4‑sulfophenyl)-2H‑tetrazolium (MTS) assay), antioxidant capacity [2,2-diphenyl-1-picrylhydrazyl (DPPH)], intracellular reactive oxygen species (ROS; 2,7-dichlorodihydrofluorescein diacetate), cytokine expression (enzyme-linked immunosorbent assay and quantitative polymerase chain reaction), and mitochondrial membrane potential (5,5′,6,6′‑tetrachloro‑1,1′,3,3′‑tetraethylbenzimidazolylcarbocyanine iodide (JC-1) assay) were assessed. Lipopolysaccharide (LPS) served as an inflammatory control, whereas S1 was used to model SARS-CoV-2-mediated neuroinflammation and mitochondrial damage.

**Results:**

All saffron derivatives showed antioxidant activity, with safranal demonstrating the strongest DPPH radical scavenging effect and the most pronounced reduction of intracellular ROS. In LPS-stimulated BV2 cells, safranal significantly decreased inducible nitric oxide synthase expression. In PBMCs, saffron compounds attenuated LPS-induced interleukin-1 beta (IL-1β) release, with safranal showing the greatest decrease. S1 increased IL-1β and tumor necrosis factor-alpha expression in BV2 microglia. Co-treatment with safranal reduced these cytokines by ∼38% and 44%, respectively. S1 induced a loss of mitochondrial membrane potential, which was effectively restored by safranal, as confirmed by JC-1 fluorescence analysis.

**Conclusions:**

These findings identify safranal as a promising neuroprotective candidate for preventing or mitigating SARS-CoV-2-associated neurological damage and other disorders involving microglial activation and mitochondrial impairment.

## Introduction

**FIGURE 1:**
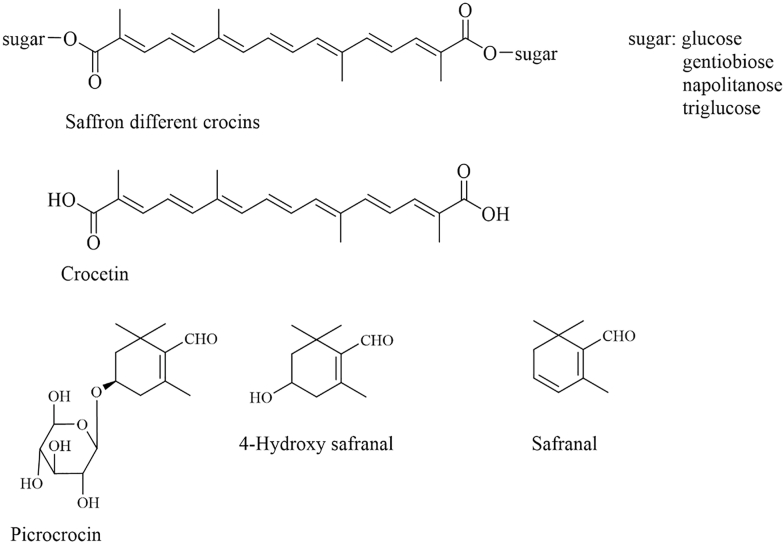
Chemical structures of the main components of saffron.

Saffron (*Crocus sativus L.*) is a spice and natural colorant that has been used for centuries. However, in recent decades, it has attracted significant scientific interest because of its pharmacological potential. In fact, saffron is used in Chinese traditional medicine to treat various diseases. The sensory properties of this spice are derived from 3 classes of compounds: crocins, responsible for the red color; picrocrocin, which imparts a bitter taste; and safranal, an aldehyde monoterpene that determines its characteristic aroma (Figure 1) [1]. The quantification of crocins, picrocrocin, and safranal is established in the International Organization for Standardization (ISO) standards for saffron and underpins the quality and grading of this spice. Saffron is among the most precious and costly spices worldwide, and what makes it even more fascinating is that not only the spice itself, but also its by-products, are rich in bioactive compounds with potential health benefits [2].

In addition to its gastronomic value, safranal has attracted attention for its multiple biological effects, including antioxidant, anti-inflammatory, anxiolytic, antidepressant, and neuroprotective activities [1]. Although safranal is essential for the typical fragrance of the spice, its natural concentration in saffron stigmas is relatively low. This intrinsic limitation makes it difficult to isolate sufficient amounts of the compound directly from plant material for experimental purposes. For this reason, pure safranal was prepared from its direct precursor 4-hydroxysafranal, obtained in turn from picrocrocin as previously reported [3].

Concurrently, crocins and other saffron carotenoids have demonstrated neuroprotective properties in in vitro and in vivo experimental models. In particular, crocin has been shown to counteract cell death induced by hypoxia and serum deprivation by promoting glutathione synthesis and reducing the accumulation of ceramide, a known mediator of neuronal apoptosis [4]. In animal models of cerebral ischemia, crocin administration resulted in a significant reduction in infarct areas, confirming a potential role in limiting neuronal damage from oxidative stress and ischemia [4]. Furthermore, current results indicate that safranal protects against oxygen-glucose-serum deprivation-induced neurotoxicity by modulating oxidative and apoptotic responses in neuronal cells derived from a pheochromocytoma of the rat adrenal medulla (PC12) [5]. These data augment extant evidence attributing beneficial effects to saffron at the cardiovascular, metabolic, and antitumor levels, delineating a phytocomplex of considerable interest for biomedical research [1].

Regarding the pharmacological properties of safranal, it displays excellent lipophilicity, high permeability, and high plasma protein binding [6]. Despite these characteristics, oral absorption in vivo was very poor, a phenomenon related to the structural integrity of safranal in simulated gastric fluid, simulated intestinal fluid, plasma, and liver microsomes [6]. Research conducted on liver microsomes has demonstrated that safranal is characterized by a high degree of chemical instability, with the capacity to undergo rapid transformations [6]. These transformations are likely to be the result of oxidation and the formation of hydroxylated derivatives [6]. The α, β-unsaturated aldehyde renders safranal particularly vulnerable to chemical and metabolic degradation, resulting in rapid loss of the molecule in both the gastrointestinal environment and following absorption. Under physiological conditions, safranal oscillates between the aldehyde form and a hydroxylated acetal form, an equilibrium that compromises its stability in gastric and intestinal fluids. Once absorbed, the situation does not improve: the compound is rapidly metabolized in the liver and degraded in plasma, significantly reducing the time it remains available in the systemic circulation. In addition to this, its strong binding to plasma proteins must be considered, which, although a characteristic common to many bioactive molecules, in the case of safranal, can limit the free fraction that is actually capable of exerting a biological effect. In summary, therefore, the chemistry of safranal creates an apparent paradox: a structure that on paper appears to have excellent pharmacokinetic properties but which, because of its instability and reactivity, significantly hinders the actual oral bioavailability of the compound [6]. Further studies are needed to determine the stable and orally absorbable form of safranal, derivatizing its aldehyde group without compromising its potency. Furthermore, the preponderance of evidence regarding neuroprotective and antidepressant effects reported in animal models is largely indirect, with a paucity of quantitative in vivo studies on the brain/plasma ratio and the ability of safranal to cross the blood–brain barrier. Safranal represents a promising compound for the treatment of neurodegenerative disorders and depression; however, its poor oral bioavailability is a major limitation. Approaches based on chemical modifications or innovative formulations, such as nanoparticles or intranasal administration, could improve its stability and absorption.

The focus on saffron has been further intensified by the emergence of the severe acute respiratory syndrome coronavirus 2 (SARS-CoV-2) pandemic. The emergence of severe acute respiratory syndrome SARS-CoV-2 in 2019 triggered a global pandemic that has affected most countries and territories. In addition to respiratory complications associated with SARS-CoV-2 disease, mounting evidence suggests a correlation between neurological symptoms and COVID-19 [7]. The extant clinical data pertaining to the current pandemic of severe acute respiratory syndrome SARS-CoV-2 suggest that ∼40% of patients infected with the virus develop neurological symptoms. In patients with long-term SARS-CoV-2 infection, chronic neuroinflammation, and neuronal damage result in a syndrome that has been described as neuro-COVID [8,9]. A plethora of studies have documented neurological symptoms in both the acute and postinfection phases, ranging from anosmia and ageusia to cognitive impairment, persistent headache, encephalopathy, stroke, and peripheral neuropathy [[9], [10], [11]]. Completely effective drugs and treatments are not recognized for this virus. The pathophysiology of neuro-COVID is intricate and appears to involve multiple mechanisms, including direct entry of the virus into the central nervous system (CNS) through the angiotensin-converting enzyme 2 receptor, endothelial dysfunction, and coagulation alterations that cause cerebrovascular events, a systemic inflammatory response with massive cytokine release, and oxidative stress processes that promote neuronal damage [12]. A study has demonstrated that the SARS-CoV-2 subunit Spike protein 1 (S1) has the capacity to induce a loss of barrier integrity and a proinflammatory response in a 3D model of the human blood–brain barrier [13], thus proposing a potential mechanism for the neurological symptoms induced by the coronavirus. It has been posited by other studies that mechanisms of SARS-CoV-2–mediated CNS dysfunction exist [14,15]. It is imperative to gain a more profound comprehension of the cellular mechanisms implicated in the neurological ramifications of SARS-CoV-2 infection, as this is instrumental in the identification of pharmacological strategies aimed at the prevention and treatment of the condition. In this scenario, the biological properties of saffron compounds are of particular relevance. Molecular dynamics simulations have demonstrated the ability of crocin, crocetin, picrocrocin, and safranal to interact with critical SARS-CoV-2 proteins, including the spike protein and the main protease. This finding suggests a possible antiviral effect [16]. Concurrently, the antioxidant and anti-inflammatory properties of saffron compounds may contribute to a reduction in neurodegenerative processes, which are characteristic of post-COVID neurological complications. The observations presented herein substantiate the mounting interest in saffron and its constituents as prospective neuroprotective and antiviral agents, particularly within the context of neuro-COVID. The objective of this study is to analyze the effects of saffron-derived compounds on immortalized murine microglial cell line (BV2) microglial cells, with particular attention to their potential role in the prevention and management of neurological effects associated with SARS-CoV-2 infection. In particular, microglia-mediated neuroinflammation has been demonstrated to play a significant role in many neurological disorders [[17], [18], [19], [20]]. Microglia, the resident immune cells of the CNS, possess the capacity to migrate, proliferate, and phagocytose. On exposure to a pathogen, microglia, which are in their “resting” state, transition to an activated state and rapidly mobilize to the site of injury to initiate an immune response. These characteristics render microglia a widely used model for the study of neuroinflammation [21,22]. This article analyzes the effects of saffron derivatives (picrocrocin, 4-hydroxysafranal, and safranal) on both peripheral blood mononuclear cells (PBMCs) and CNS microglia cells. Cells derived from raf/myc immortalized murine neonatal microglia (BV-2) will be used to study the effect of S1 protein on microglial cells. This cell line is the most frequently used substitute for primary microglia [23] and has been used for pharmacological studies, phagocytosis studies, and for many important immunological discoveries. This discovery reinforces the growing interest in saffron and its constituents as potential neuroprotective and antiviral agents. Specifically, the study shows that saffron derivatives, particularly safranal, have strong antioxidant, anti-inflammatory, and neuroprotective properties. They reduce oxidative stress, modulate peripheral inflammation, and prevent spike protein-induced mitochondrial dysfunction in microglial cells.

## Methods

### Materials

Recombinant human coronavirus SARS-CoV-2 spike glycoprotein S1 (active), purchased from Abcam (ab273068) (accession MN908947), is a SARS-CoV-2 fragment protein, in the 16 to 685 aa range, expressed in epithelial cell line from the ovary of the Chinese hamster (CHO) cells, with >90% purity. The observed molecular weight of the protein is 125 kDa. The protein is suspended in 1.74% sodium chloride, 0.82% sodium phosphate at a concentration of 1mg/mL. Lipopolysaccharide (LPS) is derived from *Escherichia coli* (Sigma) solubilized in water at a concentration of 1 mg/mL. Organic solvents were purchased from Sigma Aldrich. Nuclear Magnetic Resonance (NMR) spectra were performed on a 600 MHz Bruker Avance spectrometer. The residual proton signal of the deuterated solvent was used as an internal reference. Pure safranal was prepared from its direct precursor 4-hydroxysafranal, obtained in turn from picrocrocin as recently reported by us [3].

### Extraction of picrocrocin and synthesis of 4-hydroxysafranal and safranal

Picrocrocin was extracted from plant material collected in October 2024 in agricultural fields located in the municipality of Tradate, Province of Varese, Northern Italy (latitude: 45°42′30″ N, longitude: 8°54′27″ E, elevation: 303 m above sea level). Drying of saffron stigmas was performed at 40°C for 1 h. Until the weight was constant (24 h), finely grinded and stored at −20°C. For the extraction, 2.00 g of saffron powder residues were added to 12 mL of n-hexane, and the mixture was stirred in the dark at room temperature for 24 h. Then the organic solvent was discarded, the solid residue was dried under vacuum, and extracted with 24 mL of water (Millipore). The resulting suspension was stirred for 1 h in the dark at room temperature. Then the extract was centrifuged at 4000 × *g* for 10 min, and the supernatant was collected and dried under reduced pressure. Crude extract was purified by C18 column chromatography (H_2_O to H_2_O/MeOH 99:1) to obtain pure picrocrocin in 50% yield. ^1^H-NMR (600 MHz, CD_3_OD) δ: 10.09 (1H, s), 4.43 (1H, d, J = 7.71 Hz), 4.09 (1H, m), 3.82 (1H, d, J = 12.02 Hz), 3.67 (1H, dd, J = 4.90,12.02 Hz), 3.36 (1H, m), 3.26 (1H, m), 3.16 (1H, t, J = 7.60 Hz), 2.69 (1H, m), 2.32 (1H, m), 2.12 (3H, s), 1.88 (1H, m), 1.54 (1H, t, J= 12.10 Hz), 1.23 (3H, s), 1.21 (3H, s). ^13^C-NMR (150 MHz, CD_3_OD) δ: 193.6, 155.8, 141.3, 102.7, 78.2, 78.1, 75.3, 72.1, 71.8, 62.9, 48.4, 42.4, 36.7, 29.5, 28.1, 19.4. Biotransformation was set up by using a halo-thermophilic β-glucosidase from Alicyclobacillus acidophilus. A 5-mL reaction mixture in 4-(2-hydroxyethyl)-1-piperazineethanesulfonic acid (HEPES) buffer 50 mM, pH 7.5, containing 10 mg/mL picrocrocin (30 mM), 1 mg/mL enzyme, was left under magnetic stirring at 40°C. Complete conversion into the corresponding 4-hydroxysafranal was observed in 15 min. After extraction with ethyl acetate (EtOAc) the organic phase was collected, dried over Na_2_SO_4_, filtered, and evaporated under reduced pressure. The crude extract was purified by silica gel column chromatography (n-Hex:EtOAc, 6:4), yielding 80%. ^1^H-NMR (600 MHz, CDCl_3_) δ: 10.11 (1H, s, CHO), 3.98 (1H; br s), 2.52 (1H, dd, J = 4.82, 9.04 Hz), 2.47 (1H, dd, J = 4.82, 9.04 Hz), 2.17 (3H, s), 1.72 (2H, d, J = 12.01 Hz), 1.26 (6H, s). ^13^C-NMR (150 MHz, CDCl_3_) δ: 193.5, 154.4, 141.9, 66.0, 51.4, 46.4, 37.8, 30.8, 29.7, 21.2. Subsequent safranal was obtained by dehydration: a solution of 10 mg/mL (60 mM) of 4-hydroxysafranal in water was prepared in sealed screw cap tubes; the reaction mixture was left under magnetic stirring at 60°C. Complete conversion into the corresponding safranal was observed after an overnight reaction (>99%). After extraction with diethyl ether, the organic phase was collected, dried over Na_2_SO_4_, filtered, and evaporated under N_2_ flow. ^1^H-NMR (600 MHz, CDCl_3_) δ: 10.09 (1H, s, CHO), 6.11 (1H; quint, J = 9.31, 4.74 Hz), 5.88 (1H, dt, J = 9.31, 1.93 Hz), 2.12 (3H, s), 2.10 (1H, dd, J = 4.74, 1.93 Hz), 1.18 (6H, s). ^13^C-NMR (150 MHz, CDCl_3_) δ: 191.8, 146.9, 137.4, 134.5, 129.9, 41.0, 32.6, 26.2 (×2C), 17.7. Safranal purity, assessed by NMR analysis, was >95%. The corresponding ^1^H and ^13^C NMR spectra are provided in the supporting information.

### Cell culture

BV2 microglial cells were cultured in Dulbecco’s Modified Eagle Medium (DMEM) (GIBCO) supplemented with 10% Fetal Bovine Serum (FBS) (GIBCO), 100 U/mL penicillin and 100 U/mL streptomycin, and 2 mM L-glutamine.

### Determination of cell viability

Cell viability was measured by MTS assay (Promega). MTS [3-(4,5-dimethylthiazol-2-yl)-5-(3-carboxymethoxyphenyl)-2-(4-sulphophenyl)-2H-tetrazolium] was used according to the manufacturer’s instructions. Cells were seeded at a density of 5 × 10^3^ cells/well on 96-well plates. After the treatment, with different doses (0.05, 0.1, 0.25, 0.5, 1, and 2 mM) of picrocrocin, 4-hydroxysafranal, and safranal for 24 and 48 h, or with spike protein (0.75–1.5–3 ug/mL) for 72 h, 20 μL of the MTS solution was added to each well, and incubated for 4 h at 37°C, 5% CO_2_. The absorbance was read at 490 nm on the microplate reader GloMax Discover System (Promega). Results were expressed as the percentage of MTS reduction relative to the control. The treated cultured cells and the controls were morphologically analyzed by inspection with an Olympus CKX43 microscope.

### 2,2-Diphenyl-1-picrylhydrazyl - DPPH assay

The antioxidant power was evaluated through a 2,2-diphenyl-1-picrylhydrazyl (DPPH) assay (Bioquochem, BQC). To this end, the stock solution of extracts was diluted in phosphate-buffered saline (PBS) buffer to reach the concentration at 0.5 mM. Further, 20 μL of this dilution was diluted in 200 μl of DPPH reagent. Trolox (TX) was used for the calibration curve. The oxidation kinetics were evaluated adopting the GloMax Discover System (Promega) at 517 nm of absorbance, in a 96-multiwell plate incubated for 30 minutes. The antioxidant activity was expressed in terms of trolox equivalents antioxidant capacity.

### Evaluation of ROS generation

Intracellular reactive oxygen species (ROS) levels were measured by using 2,7-dichlorodihydrofluorescein diacetate (DCFH-DA). BV2 cells were plated in 96 well plates at a density of 5 × 10^3^/well, allowed to grow overnight, and incubated with different doses (0.05, 0.1, 0.25, and 0.5 mM) of picrocrocin, 4-hydroxysafranal, and safranal for 24 h or by LPS (0.1 μg/mL) in combination with picrocrocin, 4-hydroxysafranal, and safranal (0.05 and 0.01 mM) for 4 h. After the incubation time, the medium was replaced with PBS buffer containing DCFH-DA (200 μM) and incubated for 15 min at 37°C. At the end, PBS washes were performed to remove the probe, and the fluorescence intensity was analyzed by spectrofluorimeter (GloMax reader, Promega) with an excitation of 475 nm and an emission wavelength between 500 and 550 nm.

### PBMC isolation and measurements of inflammatory markers

Twenty milliliters of venous blood were obtained from each subject. PBMCs were isolated using the Ficoll-Hypaque isolation technique. To determine cytokine expression, 1 × 10^5^ PBMCs/mL per well were distributed into 24-well plates in the presence of LPS (1 μg/mL) in combination with picrocrocin, 4-hydroxysafranal, and safranal (0.125 mM) for 24 h. Culture supernatants were collected and stored at −80°C. IL-1β levels were measured using ELISA (Sigma). For experiments conducted on PBMCs, at least 3 independent biological replicates were used, each derived from a separate donor. This approach is recommended to ensure statistical robustness in vitro studies with primary cells. In total, 10 healthy adult donors were included (5 females and 5 males), ranging in age from 25 to 40 y, to reduce potential gender bias. All participants provided written informed consent before sample collection, in accordance with ethical guidelines and institutional approval. Controlling for interindividual variability was achieved through the implementation of uniform inclusion criteria, which excluded participants with inflammatory diseases, recent infections, or those undergoing drug therapies. The study protocol was approved by the Ethics Committee (N 08/2021, 15 September, 2021), and informed consent was obtained from all participants. This study was conducted in accordance with the Declaration of Helsinki guidelines.

### Real-time qPCR

BV2 cells (5 × 10^5^ cells/mL) were seeded into 24-well plates and treated with LPS or spike protein (3 μg/mL) alone and in combination with picrocrocin, 4-hydroxysafranal, and safranal for 4 h or 24 h (0.125–0.062 mM). After treatment, BV2 cells were used to extract total RNA, and RT-PCR was used to analyze the inducible nitric oxide synthase (iNOS), IL-1 β, and TNF-α gene. Total RNA was extracted using the High Pure RNA Isolation Kit (Roche), and 1 μg of RNA was subjected to reverse transcription using the iScript cDNA Synthesis Kit (Bio-Rad). cDNAs were amplified using the SsoAdvanced Universal SYBR Green Supermix (Bio-Rad) on a StepOne real-time instrument (applied biosystems). Gene expression was validated using homemade sequence primers for mouse iNOS, IL-1β, TNF-α, and β-actin (Table 1). Gene expression was normalized to that of β-actin. The comparative quantification method CT (2^−ΔΔCT^) was used to evaluate the relative expression levels of the RNA (StepOne software, Thermo Fisher Scientific).

**TABLE 1:** Primer sequences used for real-time PCR analysis.

Gene name	Gene symbol	Primer sequences (F:forward R:reverse)
Interleukin 1beta	IL-1β	F:5′-ATGGCAACTGTTCCTGAACTCAACT-3′ R:5′-CAGGACAGGTATAGATTCTTTCCTTT-3′
Tumor necrosis factor-alpha	TNF-α	F:5′-GCCCACGTCGTAGCAAACCAC-3′ R:5′-GGCTGGCACCACTAGTTGGTTGT-3′
Inducible nitric oxide synthase	iNOS	F:5′-GGCAGCCTGTGAGACCTTTG-3′ R:5′-GCATTGGAAGTGAAGCGTTTC-3′
Glyceraldehyde-3-phosphate dehydrogenase	GAPDH	F:5′-GCCAATTCAACGGCACAGT-3′ R:5′-AGATGGTGATGGGCTTCCC-3′

### Mitochondrial membrane potential measurement

MitoProbe JC-1 assay kit (Molecular Probes Thermofisher) is used for the measurement of mitochondrial depolarization. It is a cationic dye that exhibits potential-dependent accumulation in mitochondria, indicated by a fluorescence emission shift from red to green as the mitochondrial damages and loses its membrane. Cells were treated with spike protein (3 μg/mL) alone and in combination with safranal for 24 h. Then, the cells were washed with PBS and incubated with 2 μM of JC-1 dye for 15 min at 37°C. After the incubation time, a PBS wash was performed to remove the probe. Cells were then observed under a fluorescent microscope (Olympus IX83Life Science), excitation 488, emission 530 (green), and 590 (red). The positive control was represented to carbonyl cyanide 3-chlorophenylhydrazone, a chemical inhibitor of oxidative phosphorylation and, therefore, a disrupter of the membrane potential of the mitochondria.

### Statistical analysis

All experiments were repeated at least 3 times, and each experiment was performed in triplicates. The results are presented as mean ± SD. One-way analysis of variance was performed, followed by Dunnett’s posthoc test for significance analysis. Differences were considered statistically significant at ∗*P* ≤ 0.02, ∗∗*P* ≤ 0.002, ∗∗∗*P* ≤ 0.0005, ∗∗∗∗*P* ≤ 0.0001.

## Results and Discussion

### Cytotoxicity of saffron-derived compounds

**FIGURE 2:**
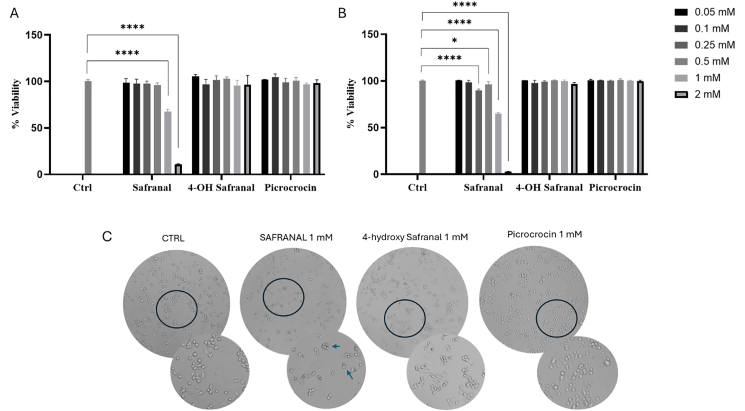
Cytotoxicity of BV2 cells treated with saffron-derived compounds. The cells were treated with picrocrocin, 4-hydroxysafranal, and safranal (0.05–2 mM) for 24 h (A) and 48 h (B). (C) Morphological analysis of BV2 treated for 48 h with picrocrocin, 4-hydroxysafranal, and safranal at 1 mM. All experiments were done in triplicate that represented as the means ± SD. Differences were considered statistically significant at ^∗^*P* ≤ 0.02, ^∗∗∗∗^*P* ≤ 0.0001. BV2; immortalized murine microglial cell line; Ctrl, control.

As a preliminary investigation, the cytocompatibility of picrocrocin, 4-hydroxysafranal, and safranal was examined in BV2 microglial cells at different concentrations and for 2 treatment times (24 and 48 h). As demonstrated by the histograms presented in Figure 2, it is evident that toxicity manifests exclusively in the presence of safranal at the elevated doses of 1 and 2 mM (Figure 2A and B). Specifically, after 48 h of treatment, cell viability was 64.88 ± 1.18% at a concentration of 1 mM safranal, whereas it dropped dramatically to 2.84 ± 0.38% at 2 mM. Cytocompatibility was also confirmed by microscopic inspection, where no morphological variation was detected in the treatments performed with the exception of the treatment with to high doses of safranal (Figure 2C). In particular, the observed morphological alterations were characterized by a marked reduction in cell body volume, accompanied by loss of normal morphology, increased cellular aggregation, and a tendency of cells to form irregular clusters, indicative of impaired adhesion (Figure 2C, arrows). These alterations are indicative of a process of cellular distress that may signal degenerative or apoptotic states. This effect is consistent with data reported in the literature, which show that exposure to safranal causes a concentration-dependent inhibition of PC12 cell viability [5]. Moreover, Safranal has been shown to reduce the viability of human epithelial cells derived from a cervical cancer biopsy cells in a concentration-dependent manner [24]. This study demonstrates that safranal exerts a targeted antiproliferative effect by disrupting the secondary structure of tubulin and interfering with the reassembly potential of microtubules [24]. Moreover, Safranal showed clear antiangiogenic activity: it inhibited hypoxia-inducible factor 1-alpha proliferation (IC_50_ ≈ 300 μM), reduced vascular endothelial growth factor (VEGF) secretion in human liver–derived cancer cell line cells, and blocked VEGF-driven angiogenesis in multiple in vitro and ex vivo assays. Safranal downregulated key angiogenesis-related proteins, including hypoxia-inducible factor 1-alpha, VEGF, VEGF receptor 2, phosphorylated Akt (protein kinase B), phosphorylated extracellular signal-regulated kinase ½, Matrix metallopeptidase 9, phosphorylated focal adhesion kinase, and phosphorylated signal transducer and activator of transcription 3 [25]. In light of the findings from the preliminary experiments, it was decided that the concentration range for subsequent experiments would be between 0.05 and 0.5 mM.

### Saffron and antioxidant activity in BV2 cells

**FIGURE 3:**
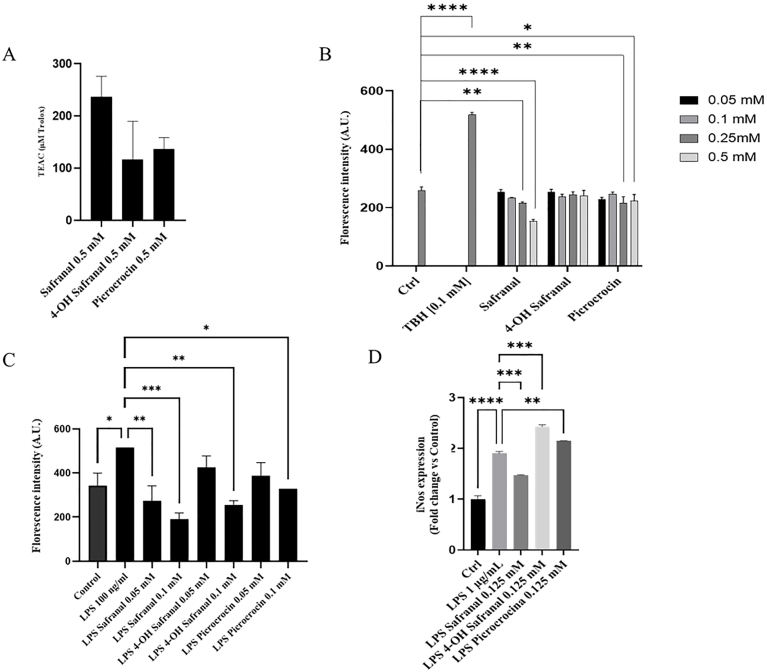
Antioxidant activity and ROS mitigation in BV2 cells. (A) Histogram of the DPPH test of saffron-derived compounds. (B) Histogram of DCFH-DA of intracellular ROS levels after treatment with saffron-derived compounds. (C) Histogram of DCFH-DA in acute oxidative stress induced by LPS for 4 h alone or in combination with saffron-derived compounds. The TBH as used as a positive control. (D) iNos expression levels after treatment with saffron-derived compounds in combination with LPS. Data in the histograms are presented as the mean of 3 different experiments. Differences were considered statistically significant at ^∗^*P* ≤ 0.02, ^∗∗^*P* ≤ 0.002, ^∗∗∗^*P* ≤ 0.0005, ^∗∗∗∗^*P* ≤ 0.0001. BV2; immortalized murine microglial cell line; Ctrl, control; DCFH-DA, 2,7-dichlorodihydrofluorescein diacetate; DPPH, 2,2-diphenyl-1-picrylhydrazyl; iNOS, inducible nitric oxide synthase; LPS, lipopolysaccharide; ROS; reactive oxygen species; TBH, tert-butyl hydroperoxide; TEAC, trolox equivalents antioxidant capacity.

The 2,2-diphenyl-1-picrylhydrazyl (DPPH) test was performed, a method that is widely recognized in scientific research as it provides a reliable means of assessing the antioxidant properties of various substances and their ability to neutralize free radicals. The findings of the study indicated that all 3 saffron derivatives exhibited antioxidant activity, with safranal demonstrating the highest level of antioxidant activity (Figure 3A). Moreover, to validate this effect, the present study examined the potential of saffron-derived compounds to decrease intracellular levels of ROS. The results of the study demonstrate that treatment with saffron-derived compounds, in particular for safranal compound, is associated with a significant reduction of intracellular ROS (Figure 3B). The reduction of intracellular ROS accumulation, for the safranal, was concentration-dependent, with the concentrations ranging from 0.1 to 0.5 mM (Figure 3B). In particular, the presence of safranal at a concentration of 0.5 mM has been shown to reduce the amount of ROS measured indirectly with the DCFH-DA assay by ∼50%. The tert-butyl hydroperoxide was used as a positive control (Figure 3B). To demonstrate the capacity of safranal derivatives to reduce oxidative stress induced by external agents, an experiment was conducted in which microglial cells were treated with LPS, thus simulating an inflammatory stimulus (Figure 3C). The results demonstrated that all saffron-derived compounds exhibited the capacity to reduce oxidative stress induced by LPS treatment, with safranal administration eliciting the most pronounced effects (reduction of ∼60% compared with LPS at 0.1 mM safranal concentration). To verify this result, levels of iNOS were analyzed. iNOS is responsible for the production of substantial amounts of nitric oxide (NO) and is induced in macrophages and microglia in response to inflammatory mediators such as LPS. As demonstrated in Figure 3D, the histogram reveals that administration of LPS resulted in increased expression of the iNOS gene, indicative of an ongoing oxidative and inflammatory process. Treatment with safranal alone, in contrast to picrocrocin and 4-hydroxysafranal, resulted in a reduction in these levels (∼25% reduction compared with LPS), suggesting that safranal has the ability to mitigate nitrosyl oxidative stress (Figure 3D). These results are consistent with data reported in the literature on PC12 neuronal cells treated with safranal after an oxygen-glucose-serum deprivation insult [5]. Although the exact reason for its antioxidant behavior is not clear, this feature is probably due to the extended conjugation present in its structure, which quenches singlet oxygen in a reaction that involves the transfer of excitation energy from ^1^O_2_ to safranal molecule. Therefore, the conjugated aldehyde could provide a reactive chemical moiety, capable of mediating electron transfer, scavenging free radicals, or quenching singlet oxygen [26].

### Peripheral anti-inflammatory effect of saffron-derived compounds

**FIGURE 4:**
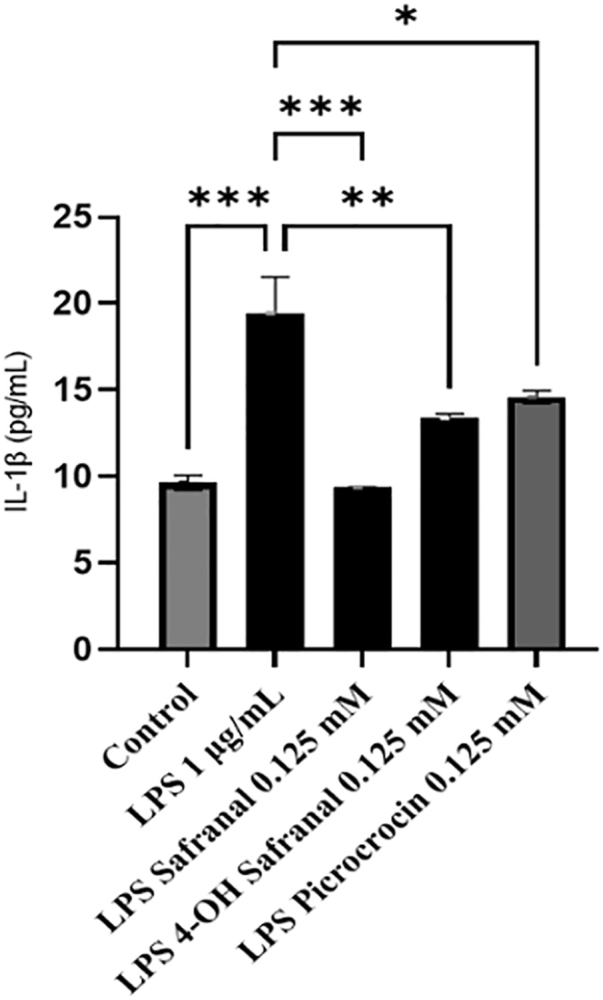
Anti-inflammatory effect of saffron-derived compounds. (A) Schematic representation of the experimental procedure. (B) ELISA quantification of IL-1β levels in supernatants of untreated PBMCs (control), treated with saffron-derived compounds and LPS. Data in the histograms are presented as the mean of 3 different experiments. Differences were considered statistically significant at ^∗^*P* ≤ 0.02, ^∗∗^*P* ≤ 0.002, ^∗∗∗^*P* ≤ 0.0005. PBMC, peripheral blood mononuclear cells; LPS, lipopolysaccharide.

Furthermore, the study sought to test whether saffron-derived compounds could generate anti-inflammatory effects at the peripheral level. To achieve this, PBMCs were extracted from healthy volunteers and treated with saffron-derived compounds and LPS, an inflammatory response activator (positive control). As demonstrated by the histogram presented in Figure 4, saffron-derived compounds were able to reduce the LPS-induced inflammatory response by resulting in a decrease in IL-1β levels (safranal reduces 1β levels by 52%, 4-hydroxysafranal by 32%, and picrocrocin by 25% compared with LPS). Among the saffron-derived compounds, safranal administration elicited the most pronounced effects.

### Effect of the S1 on microglia cell viability

**FIGURE 5:**
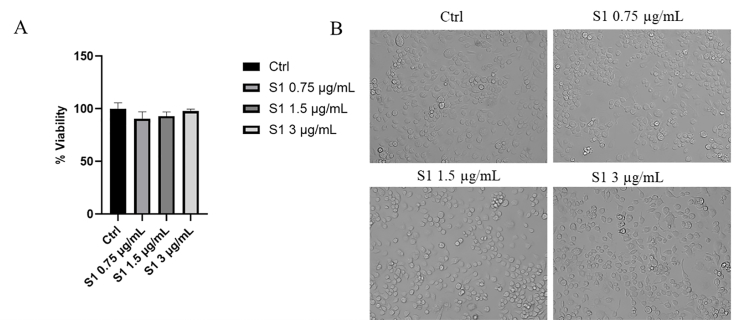
Effect of SARS-CoV-2 spike protein on cell viability. (A) Histogram of the effect of SARS-CoV-2 spike protein on microglia cell viability expressed as % cell viability compared with untreated control. (B) Morphological analysis of the effect of spike protein on microglia at the highest concentration. Data in the histograms are presented as the mean of 3 different experiments. Ctrl, control; S1, spike protein 1; SARS-CoV-2, severe acute respiratory syndrome coronavirus 2.

As a preliminary step in the research, the effect of the S1 on microglia cell viability was evaluated at varying doses. As demonstrated in Figure 5A, a histogram illustrating the viability of BV2 cells treated with varying doses of the spike protein, for 72 h, indicates that the treatment did not induce substantial cellular toxicity at the exposure times and concentrations used in the in vitro tests. This effect is also confirmed by light microscopy analysis, where no changes in cell morphology are observed (Figure 5B). These data are consistent with previous studies demonstrating that no toxic effects on microglia cells are evident [27].

### Protective effects of safranal against SARS-CoV-2 inflammation and mitochondrial dysfunction in BV2 cells

**FIGURE 6:**
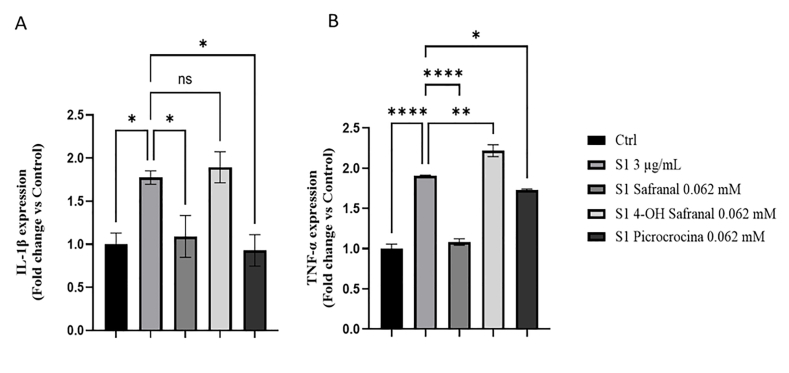
Effect of safranal and SARS-CoV-2 spike protein 1 (S1) on proinflammatory mediators in BV2 cells. (A) IL-1β and (B) TNF-α mRNA expression. Data in the histograms are presented as the mean of 3 different experiments. Differences were considered statistically significant at ^∗^*P* ≤ 0.02, ^∗∗^*P* ≤ 0.002, ^∗∗∗∗^*P* ≤ 0.0001. BV2, immortalized murine microglial cell line; Ctrl, control; SARS-CoV-2, severe acute respiratory syndrome coronavirus 2.

In line with previous studies, exposure to the S1 subunit of the SARS-CoV-2 spike protein led to a significant increase in the production of proinflammatory mediators, such as IL-1β and TNF-α, in BV2 cells [28]. Concomitant administration of saffron-derived compounds, however, substantially attenuated this proinflammatory response, as demonstrated by reduced cytokine expression levels (safranal reduces the expression levels of IL-1β by 38% and TNF-α by 44%) (Figure 6 A and B). In particular, a greater effect for both cytokines was observed after treatment with safranal (Figure 6 A and B). These results suggest that safranal has a significant anti-inflammatory effect and can counteract S1-induced inflammation in microglial cells. These data support its potential as a neuroprotective agent against spike protein-mediated inflammatory damage.

In view of the established superiority of safaranal in terms of antioxidant (Figure 3) and anti-inflammatory (FIGURE 4, FIGURE 6) properties when compared with other components, the present study concentrated on investigating its capacity to counteract the deleterious effects of the spike protein on mitochondrial dysfunction in microglia cells BV2. It is acknowledged that inflammation and mitochondrial dysfunction are interconnected. Nuclear factor kappa-light-chain-enhancer of activated B cells (NF-κB) activation and the production of proinflammatory cytokines (TNF-α, IL-1β) induce oxidative stress, alter mitochondrial membrane permeability, and promote the release of cytochrome C, amplifying the apoptotic cascade [29,30]. Conversely, mitochondrial damage has been demonstrated to increase ROS generation, which in turn activates NF-κB, thereby creating a positive feedback loop between neuroinflammation and neuronal apoptosis [29,31,32].

**FIGURE 7:**
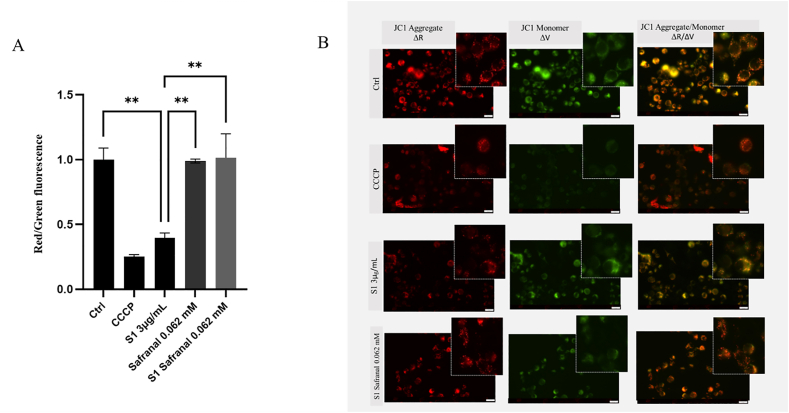
Effect of SARS-CoV-2 spike protein and safranal on mitochondrial dysfunction. (A) Histogram of 5,5’,6,6’-tetrachloro-1,1’,3,3’-tetraethylbenzimidazolylcarbocyanine iodide (JC-1) fluorescence intensity of BV2 cells treated with Spike protein alone or in combination with safranal. (B) Representative fluorescence microscopy images. Data in the histograms are presented as the mean of 3 different experiments. Differences were considered statistically significant at ^∗∗^*P* ≤ 0.002. BV2, immortalized murine microglial cell line; CCCP, carbonyl cyanide 3-chlorophenylhydrazone; Ctrl, control; S1, spike protein 1; SARS-CoV-2, severe acute respiratory syndrome coronavirus 2.

It has been widely demonstrated that the virus responsible for severe acute respiratory syndrome is capable of inducing mitochondrial dysfunction and activating the intrinsic mitochondrial-dependent apoptotic pathway, resulting in microglial cell apoptosis [27]. In the initial phase of the present study, an assessment was conducted to ascertain whether the SARS-CoV-2 spike protein had the capacity to compromise mitochondrial integrity. This assessment was undertaken using a specific mitochondrial membrane potential assay. The results demonstrated that exposure to the spike protein resulted in a significant decrease in membrane potential (red/green) (Figure 7). As demonstrated in the histogram, the spike protein-induced a significant reduction, ∼60%, in mitochondrial potential in BV2 cells, thereby confirming its direct role in inducing mitochondrial damage (Figure 7A). Finally, BV2 cells were treated with the spike protein in combination with safranal. The analysis demonstrates that safranal effectively restores impaired mitochondrial function. (Figure 7A). The aforementioned effect was confirmed by means of an investigation that employed fluorescence microscopy (Figure 7B).

The findings of this study are consistent with those reported by Yan et al. [33], which suggest that safranal exerts neuroprotective effects through a multitarget modulation of mitochondrial and inflammatory pathways. In particular, the safranal inactivates glycogen synthase kinase-3 beta (GSK-3β) by means of Ser9 phosphorylation and reduction of Tyr216 phosphorylation, thus limiting its proapoptotic activity. GSK-3β, when active in mitochondria, has been demonstrated to promote the loss of membrane potential, increased oxidative stress, and the release of cytochrome C, as well as perturbed mitochondrial morphology. These events have been shown to activate the intrinsic apoptosis cascade and contribute to neuronal degeneration [30,33]. Concurrently, safranal exerts a suppressive effect on the phosphorylation of NF-κB p65 and Inhibitor of kappa B alpha, thereby impeding NF-κB nuclear translocation and curtailing the production of proinflammatory cytokines such as TNF-α and IL-1β. These are closely associated with mitochondrial dysfunction and the exacerbation of neuronal damage [33]. Furthermore, the restoration of the B-cell lymphoma 2/Bcl-2-associated x protein ratio and the reduction in the expression of Bcl-2 homologous antagonist/killer, cytochrome C, and active caspases, after safranal treatment, confirms its ability to block the mitochondrial apoptotic pathway [33,34]. The findings of this study provide a robust foundation for the further investigation of safranal as a potential therapeutic agent, with the potential to simultaneously protect mitochondria and suppress neuroinflammation. A recent study utilizing cerebral ischemia models demonstrated that safranal increases sirtuin 1 (SIRT1) expression, thereby promoting neuronal survival, neurogenesis, and angiogenesis. Furthermore, SIRT1 silencing has been demonstrated to abolish safranal’s neuroprotective effects, thus indicating that the SIRT1 pathway is essential for the action of safranal [35]. SIRT1 is an NAD^+^-dependent deacetylase that regulates numerous processes, including metabolism, oxidative stress, and longevity. The activation of this process leads to the deacetylation of peroxisome proliferator-activated receptor gamma coactivator 1-alpha (PGC-1α), thereby increasing its activity as a transcriptional coactivator and stimulating mitochondrial biogenesis [36,37]. PGC-1α is regarded as the master regulator of mitochondrial biogenesis and the response to oxidative stress. The regulation of this process is orchestrated by SIRT1 and AMP-activated protein kinase, with the capacity to engage with nuclear factors to enhance the expression of mitochondrial genes [31]. Moreover, safranal ameliorates renal damage, inflammation, and podocyte injury in membranous glomerulonephritis by upregulating SIRT1 and inhibiting NF-κB signaling [38]. However, further investigations are required to clarify whether safranal activates the SIRT1–PGC-1α axis, thus providing a more comprehensive understanding of its effects on mitochondrial function.

The extant literature suggests that the majority of clinical studies employ standardized saffron extracts, comprising a mixture of bioactive compounds such as crocin, crocetin, and safranal, rather than isolated compounds. Consequently, although safranal is present in these formulations, it has not yet been evaluated individually in human clinical trials. The most recent reviews corroborate this assertion: clinical data exclusively pertain to saffron extracts, whereas no published clinical studies are available for pure safranal. The efficacy of the latter is thus based solely on preclinical results obtained in animal and cellular models [39]. A notable example is a randomized, controlled trial published in Frontiers in Nutrition in 2021, which evaluated the efficacy of a saffron extract in improving mood and reducing stress in adults with subclinical symptoms of anxiety and depression. The results demonstrated an enhancement in depression and anxiety scores; however, the trial did not concentrate on isolated safranal, but rather on the complete saffron extract [40]. In parallel with experimental research, safranal has been the subject of numerous international patents, exploiting its pharmacological and cosmetic properties. Among the most notable applications are formulations for the treatment of depression and mood disorders (WO2015124318), satiety agents for obesity management (US20100028464), combination therapies for the prevention and treatment of liver cancer (AU2019264659A1, JP2020132625A), and compositions to improve cognitive function (WO2021209455A1). Other patents concern the use in the management of premenstrual syndrome (WO2020229412A1), and even as an antimicrobial agent (US9707203B1). The data presented herein corroborate the profound industrial interest in safranal. However, they also highlight a significant gap in the extant literature, namely the paucity of clinical studies that would serve to validate its efficacy and safety in human subjects. Consequently, future research should concentrate on well-designed clinical trials with the capacity to translate promising preclinical evidence into concrete therapeutic applications.

In conclusion, the findings of this study demonstrate that saffron derivatives, particularly safranal, possess substantial antioxidant, anti-inflammatory, and neuroprotective properties. The aforementioned substances have been demonstrated to reduce oxidative stress, modulate the peripheral inflammatory response, and prevent mitochondrial dysfunction induced by the spike protein in microglial cells. These results lend support to the hypothesis that saffron compounds may represent a novel therapeutic approach for the prevention and management of neurological complications related to SARS-CoV-2, as well as other neurodegenerative conditions characterized by neuroinflammation and mitochondrial damage. However, further in vivo studies and clinical trials will be needed to confirm the translational relevance of these data and define their potential therapeutic use.

## Author contributions

**AG**: Conceptualization, methodology design, supervision of experimental workflow, preparation of figures and tables, graphical layout. **MLC**: Resources, Investigation, data curation, formal analysis, Visualization, and refinement of manuscript presentation. **MB**: methodology design, technical support for analytical procedures. **LP**: sample preparation, statistical analysis, and contribution to interpretation of findings. **FN**: Data acquisition, statistical analysis, and contribution to interpretation of findings. **SD**: critical revision of manuscript for intellectual content. **GB**: Resources, technical support for analytical procedures, Visualization, and refinement of manuscript presentation. **PP**: Writing—review and editing, synthesis of results, coordination of collaborative inputs, and final approval of the version to be published, funding acquisition. **AP**: drafting of initial manuscript sections, critical revision of manuscript for intellectual content. **DN**: Supervision, validation, and final approval of the version to be published, funding acquisition.

## Data availability statement

Data described in the manuscript, code book, and analytic code will be made publicly and freely available without restriction.

## Funding

This research was supported by the project Emerging Infectious Diseases One Health Basic and Translational Research Actions addressing Unmet Needs on Emerging Infectious Diseases, INF-ACT, Spoke 1 (P. P.) and Spoke 5 (D.N.). Project number PE00000007, CUP B53C20040570005.

## Conflict of interest

The authors report no conflicts of interest.
