# Supplementary material for: Protective Effects of Safranal Against Spike Protein-Induced Mitochondrial Dysfunction and Inflammation in Peripheral and Central Immune Cells
Source: Curr Dev Nutr. 2025 Dec 25;10(1):107629. doi: 10.1016/j.cdnut.2025.107629 (PMC12853053; doi:10.1016/j.cdnut.2025.107629)
Supplement: Multimedia component 1 [file mmc1.docx]

*Supplementary materials*

Protective Effects of Safranal Against Spike Protein–Induced Mitochondrial Dysfunction and Inflammation in Peripheral and Central Immune Cells

Picone et al.

**Table of contents**

**^1^H-^13^C NMR spectra of safranal**

**
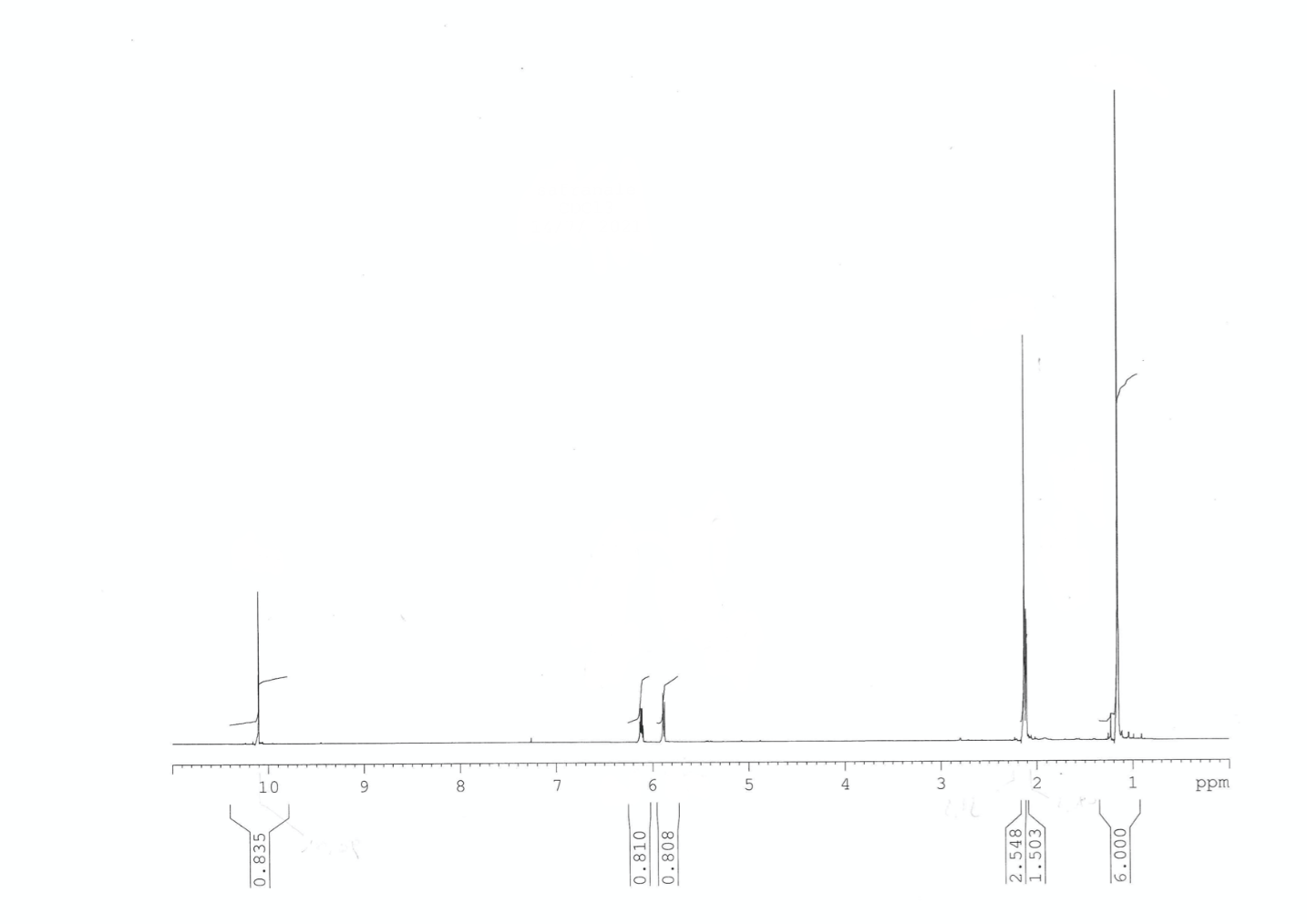
**


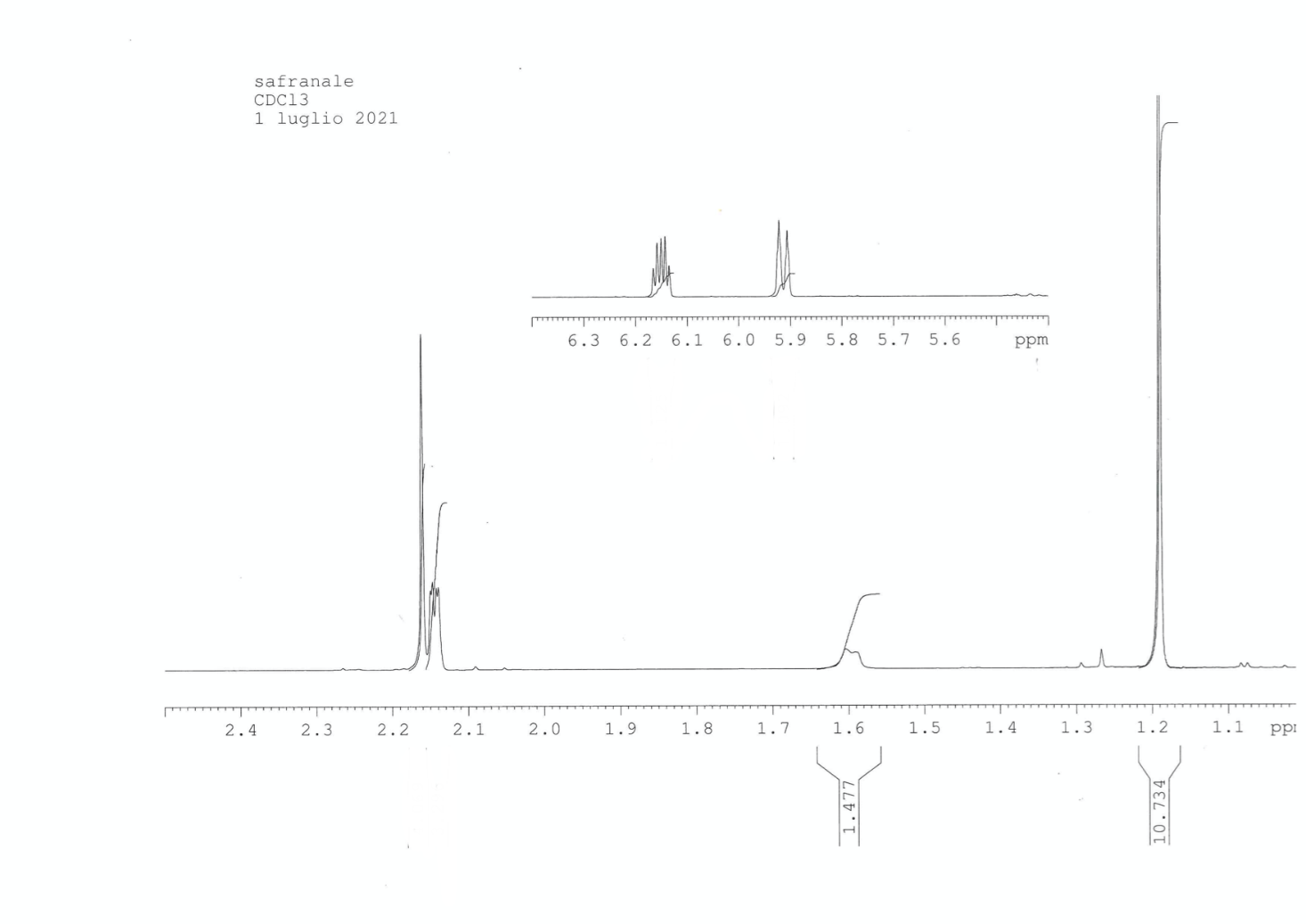

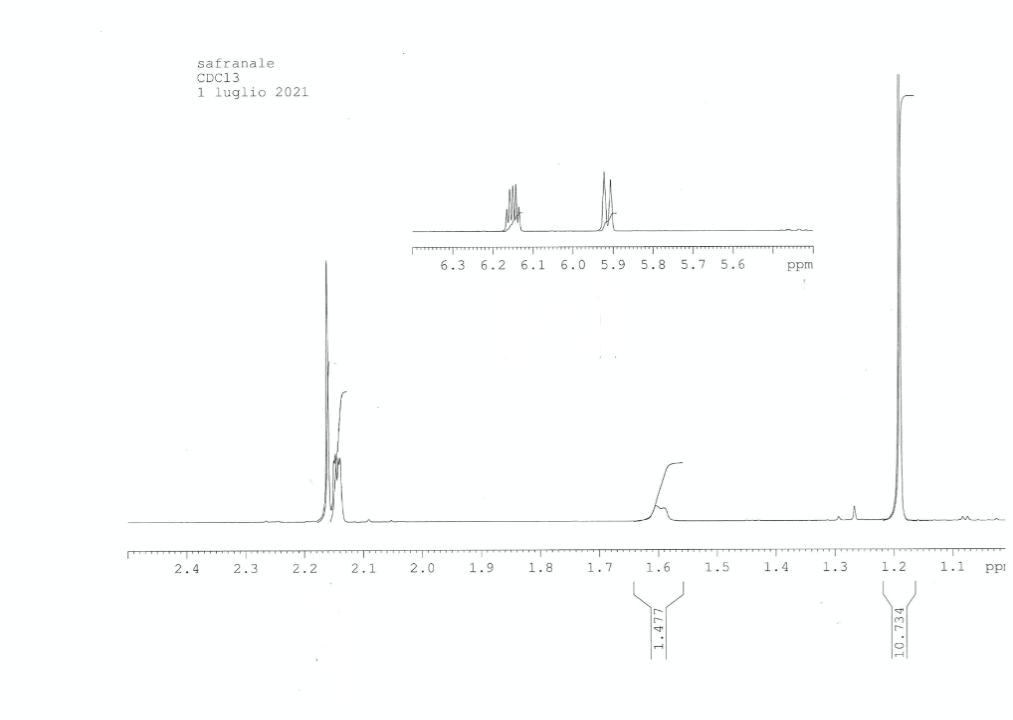


**Figure S1.** ^1^H-NMR of safranal (CDCl_3_)

**
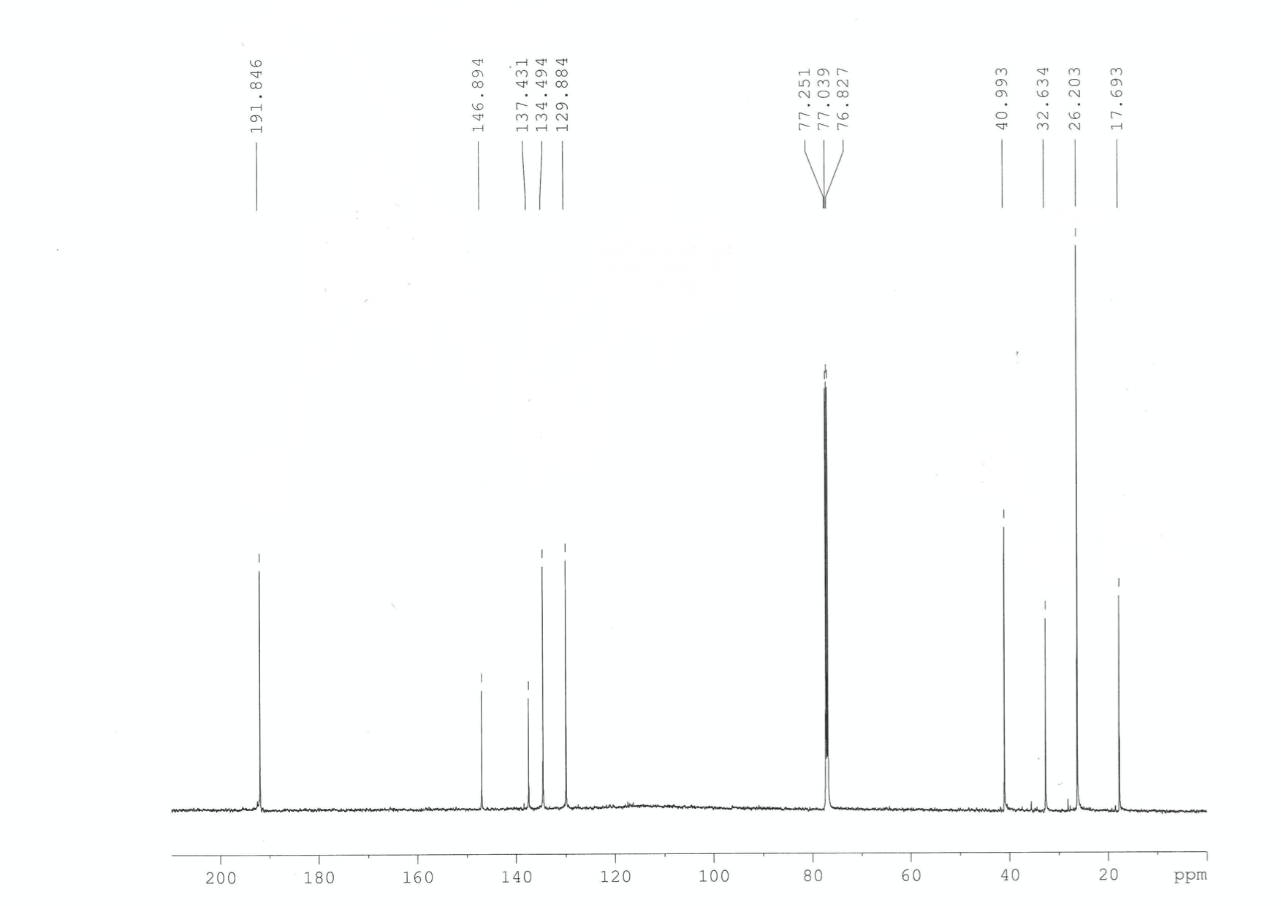
**

**Figure S2.** ^13^C-NMR of safranal (CDCl_3_)
